# Complementary and alternative medicine use in self-management of diabetes: A qualitative study of patient and user conversations in online forums

**DOI:** 10.1007/s11096-022-01469-6

**Published:** 2022-09-22

**Authors:** Abdulaziz Saud Alzahrani, Sheila M. Greenfield, Vibhu Paudyal

**Affiliations:** 1 School of Pharmacy, College of Medical and Dental Sciences, Sir Robert Aitken Institute for Medical Research, University of Birmingham, Edgbaston, Birmingham, B15 2TT UK; 2 Institute of Applied Health Research, University of Birmingham, Birmingham, UK

**Keywords:** Complementary and alternative medicine, Diabetes mellitus, Online forums

## Abstract

**Background:**

An important part of diabetes self-management includes discussing and seeking informal advice from others.

**Aim:**

This study aimed to explore beliefs and experiences of patients in relation to their use of CAM in diabetes through the use of data from online patient forum discussions.

**Method:**

Google search engine was used to identify relevant web-based online discussion forums in English language focussing on CAM use (including herbal and other unorthodox therapies) in diabetes and posted by either patients or carers. No date limit was imposed. A qualitative content analysis was adopted for analysis.

**Results:**

Twenty-two online forums containing 77 threads with 1156 posts and replies were identified. Seven major themes emerged from the data analysis including: patient beliefs regarding CAM use, perceived effectiveness and safety of CAM, evidence base and information seeking. Patients used online forums to seek information about the benefits, side effects and share positive and negative experiences of CAM use. Feeling stressed, frustrated or overwhelmed with diabetes and prescribed medications was often linked to their decisions to use CAM. They described that healthcare professionals were often unaware or unable to help in regards to their queries around CAMs.

**Conclusion:**

Patients with diabetes use online forums to share information regarding CAM use. There is a scope for professional societies, patient charities and health systems to offer such online platforms to promote rationale use of CAM, provide evidence-based information to patients and alleviate fears and concerns around diabetes and prescribed medicines.

## Impact statements

- Patients with diabetes use online forums to share their beliefs, experiences effectiveness and harm from CAM use in diabetes.
- Lack of effective patient provider communications often leads patients to resort to online forums to find and share CAM related information.
- The findings could be utilized by healthcare providers to further engage with the patients and promote rational self-care practices including the use of CAM.

## Introduction

Self-management practices are linked to disease management and clinical outcomes in patients with diabetes [1]. Self-management approaches include but are not limited to good diet, physical activities and monitoring of blood glucose [2]. Health literacy, health beliefs, self-efficacy, social support and socio-economic factors and social support are some of the key factors associated diabetes related outcomes [3]. Research suggests that patient self-management practices can often be suboptimal and have the potential to benefit from targeted interventions focused on improving knowledge and understanding of disease, prescribed treatments and importance of follow up and care [4]. Digital applications that incorporate evidence based and behaviour change model informed interventions have also been shown to promote diabetes self-management [5].

In addition to conventional medicines prescribed in healthcare settings, it is estimated that 51% of patients with diabetes use CAM as a form of diabetes self-management either as a substitute or add on to their prescribed treatments [6]. Diabetic patients use a variety of CAM types including herbal medicine, homeopathy, acupuncture, Ayurveda, yoga, massage, chiropractic and meditation. A recent systematic review reported a total of 37 types of CAM and 223 types of herbs used by diabetic patients for diabetes self-management; however, up to 2/3^rd^ patients admit that they do not disclose their CAM use to their healthcare professionals, [6] suggesting suboptimal communications between healthcare professional and patient communications regarding CAM use.

Healthcare professionals provide formal advice about diabetes self-management but patients also seek informal advice and information to help them with the management of diabetes [7]. Patients’ health information seeking behaviours have changed during the digital age contributed to by the increased availability of health information online and access to mobile technology. Online health information sources include for example websites, articles and online forums and it has been suggested that health information is one of the most searched topics online [8]. Accessibility, convenience and speed have been suggested as the main reasons that make online sources more appealing to patients [9]. Patients also exchange health information, share experiences and provide advice to one another through online forums and discussion boards [10]. Since the COVID-19 pandemic began, information seeking online has been suggested to be on the rise due to lack of face-to-face appointments and people spending more time online during lockdown [11].

Online discussion boards or online forums usually work by allowing forum users to create a post or a blog. The post can be a question, a story, an opinion or any other thought. The posts can be replied to by other forum users. The original post and the replies form what is called a thread. Some forums require a membership to post or reply to posts, others do not require membership and are available on public domains.

In formal research settings, participants may be more constrained as to what they say due to social desirability bias and therefore might not allow participants to fully and sincerely express their views and opinions [12]. Online forums hence allow exploration of patient perspectives which may not have been reported in previous studies conducted in a formal research setting. In addition, content validity of research tools is critical to obtaining reliable data in research studies.

Patient online forums and discussion boards are being recognized as an important source of ‘natural’ data and exploring them for patients’ experiences has become a method used in medical research. For example, a previous study that aimed to understand patient perspectives of epilepsy treatment analyzed user postings in three online support groups [13]. The study concluded that analyzing discussions in online forums can help guide effective provider-patient communication.

Another study aimed to explore patient perspectives on cancer chemotherapy [14]. A total of 11 related online forums were analyzed which showed that quality of life was impacted by isolation and depression as a result of the side effects that patients were previously not warned about.

A recent systematic review suggested that the internet is the main source of information for diabetic patients [15]. Information shared online between forum users is likely to play a role in shaping patients’ beliefs regarding using CAM for diabetes management [6]. However, there is a lack of research that addresses this and therefore, a study design that uses novel and non-traditional information sources would be valuable to offer patient oriented and evidence-based information in regards to CAM use in diabetes [10].

### Aim

This study aimed to explore beliefs and experiences of patients in relation to their use of CAM in diabetes through the use of data from online patient forum discussions.

### Ethics approval

The study was conducted in accordance with the British Psychological Society’s Ethics guidelines for internet-mediated research and the Association of Internet Researchers’ Internet Research [16, 17]. Ethics approval for the study was granted by the University of Birmingham Science, Technology, Engineering and Mathematics Ethical Review Committee, ethical approval number No. ERN_20-1431 on 28 September 2020.

## Method

An internet search for related forums and discussion boards was conducted using Google search engine. Google® is known to have dominated over 90% of the search engines’ market for more than 10 years, and therefore it is most likely to yield the most comprehensive results from our searches [18]. There is no consistent definition of CAM. Therefore, search terms were compiled from the results of a previous systematic review of global prevalence of CAM use and the most popular CAM types among patients with diabetes previously conducted by the authors [6]. The most commonly cited CAM types used by patients with diabetes identified by the systematic review were: (acupuncture, homeopathy, religious and spiritual healing, Ayurveda, yoga, massage, chiropractic, meditation and herbal medicine) as well as the most commonly used herbs (Aloe vera, cinnamon, fenugreek, black seed and ginger) [6]. The above terms were combined with the words “diabetes” and “forum". The search settings were set to rank the results based on relevancy to the search terms rather than the most recent results in order to facilitate the manual search for relevant forums as the search results were arranged by showing the most relevant results first and therefore could be accessed one by one until all relevant results are accessed. Google® search engine was set for the outputs to be displayed as per ‘relevance’ to the search terms. Stopping criteria were set as 10 search results in a row that did not meet the inclusion criteria. The forum search was conducted in the period between 23 September 2021 and 17 November 2021 and was restricted to forums that were available in the English language.

A forum was deemed relevant the discussion was focused on CAM use by patients with diabetes. Posts that appeared to be written by patients with diabetes (both type 1 and type 2) or individuals who are directly involved in the care of patients with diabetes, e.g. families and carers, were included. Only posts in forums that did not require membership to access their content were included as these posts are available on public domains where there is likely no perception and/or expectation of privacy by the forum users.

### Data analysis

A qualitative content and thematic analysis was adopted for the analysis of the posts to allow the data to determine the emerging themes [19]. This is an approach to analysis that is both objective and systematic in terms of recognizing specified features in textual and non-quantitative data. It is mostly used to analyze content in the field of communications by sectioning parts of the communications that are relevant to the focus of the study and assigning the codes for the resulting sections in order to identify emerging themes [20].

All threads that were deemed relevant based on content were extracted from included forums. Relevant posts were defined as those where reasons for using CAM by patients with diabetes, experiences of CAM use for diabetes or concerns raised from using CAM for diabetes management were discussed.

Extracted posts were coded based on their content. If a single post contained more than one concept, the post was segmented accordingly and each resulting segment was coded independently. Similar segments that were coded with the same code were grouped and brought together and a subtheme was identified. Sub-themes that described a common phenomenon were labelled as ‘themes’. Selection of posts was performed independently, emerging codes discussed and the final coding frame agreed by all three authors (AA, VP and SG).

## Results

The search identified 22 forums containing 77 threads with 1156 posts and replies. Following thematic content analysis on the extracted posts, 359 quotes were identified as relevant to the study objectives (Table 1) [21].

**Table 1:** List of Forums with the numbers of extracted posts

Forum name		Number of extracted posts
Total		359
1	www.diabetesdaily.com	135
2	forum.tudiabetes.org	71
3	www.diabetes.co.uk	46
4	abchomeopathy.com	13
5	forum.jdrf.org	10
6	www.shareayurveda.com	8
7	www.researchgate.net	7
8	www.diabetesforum.com	6
9	blog.resonanceschoolofhomeopathy.com	6
10	www.quora.com	6
11	www.homesteadingtoday.com	6
12	forum.diabetes.org.uk	5
13	forums.childrenwithdiabetes.com	5
14	www.vipassanaforum.net	5
15	www.internationalskeptics.com	5
16	www.drjoedispenza.net	4
17	healthunlocked.com	4
18	www.r2iclubforums.com	4
19	health.ccm.net	4
20	www.bogleheads.org	3
21	permies.com	3
22	www.city-data.com	3

Seven main themes, each with their respective subthemes, were identified from the extracted data (Table 2). The first theme explained the attitudes, feelings and experiences of CAM use by diabetic forum users. The second theme illustrated the information seeking behaviors by forum users about CAM. The next three themes illustrated efficacy, evidence of effectiveness and safety concerns about the use of CAM for diabetes management. The next theme showed patients’ views on CAM use as an alternative or additional therapy. The last theme addressed cost- related factors influencing users to resort to CAM. The number of quotes that made up each subtheme along with the related CAM type are listed in Table 2.

**Table 2:** Emerging themes,subthemes and frequency of posts

Themes	Sub-themes	Frequency of posts													
		Herbal	Aloe Vera	Cinnamon	Ginger	Fenugreek	Black seed	Homeopathy	Acupuncture	Religious	Ayurveda	Yoga	Massage	Chiropractic	Meditation
Attitude and feelings toward CAM use	Forum users express how the feel about their diabetes	2	1	0	1	0	0	1	0	3	0	1	0	1	2
	Decision making process	3	1	1	0	0	1	1	2	1	1	1	1	2	3
	Forum users’ attitude toward the use of CAM for diabetes	32	4	5	0	1	0	7	5	5	14	5	2	6	5
	Experiences associated with conventional medicines as a factor in forum users’ perceptions of CAM	8	0	0	0	0	0	3	0	0	2	0	0	0	0
	Forum users convey their healthcare providers’ attitude toward CAM	3	0	1	0	0	0	0	1	0	0	1	0	2	0
Seeking other users’ experience with using CAM for treatment of diabetes	Seeking information	9	3	1	2	1	1	3	3	0	1	1	2	0	5
	Seeking or giving instructions about how to ‘properly’ use CAM	4	0	3	1	1	0	6	1	1	1	0	0	0	3
Perceived effect of CAM	Experience of CAM use	25	4	10	3	4	2	4	7	0	3	6	2	2	7
	Forum users experience an effect of CAM on diabetes while using CAM for other reasons	0	0	0	0	0	0	0	0	0	0	0	2	1	0
Perception about evidence base of CAM effectiveness	Forum users’ views on scientific studies that about the effectiveness of CAM for diabetes	9	0	0	0	1	0	1	2	0	3	0	0	0	0
	Forum users giving explanations to CAM effectiveness	2	2	0	0	4	0	4	0	0	1	6	3	0	2
Forum users' perception on CAM safety	Perception on CAM and/or conventional medicine safety	7	0	0	0	0	0	0	1	0	0	0	1	0	0
	Using online forums to discuss CAM side effects versus conventional medicines	4	3	1	0	0	0	0	1	0	0	0	2	0	0
CAM as an additional or replacement treatment	Forum users' use or views on CAM as a complementary Therapy	4	0	0	0	0	0	1	0	0	0	0	0	0	0
	Forum users' use or views on CAM as an alternative Therapy	5	1	0	0	0	0	1	0	0	0	0	0	0	0
Economic issues around the use of CAM for the management of diabetes	Access and cost related issues from the perspective of forum users	2	0	0	0	0	0	0	0	0	0	0	0	0	1
	Forum users’ perspective on the role of corporations on CAM recognition of CAM by medical insurers	4	0	0	0	0	0	1	0	0	1	1	0	0	0

### Attitude and feelings toward CAM use

#### Forum users express how they feel about their diabetes

Forum users used online forums to convey how they felt about their diabetes and expressed the feelings that they were prepared to take any necessary steps to cure their diabetes. Patients were especially frustrated because they felt that every state of mind they are in would affect their blood glucose levels, i.e., being happy or sad.

“Type 1 diabetes is one of the issues I have that I would like get rid of but just now starting to try and figure out a way to go about it. Anything we might be able to help each other or anyone else with would be great.” (A thread about Meditation).

“Basically every system that affects your bg when the weather changes or you are upset or happy… Sadly, weird things affecting our bg is the world we live in” (A thread about Herbal Medicine).

#### Decision making process

Feeling stressed, frustrated or overwhelmed about managing diabetes was linked to forum users’ decisions to use CAM. Forum users also posted about hearing about a CAM type that is beneficial for diabetes and then conducting online searches on it. The information they found online was enough for them to try using CAM for diabetes.

“In general, mindfulness can be a useful tool to help increase our ability to tolerate the stress of managing diabetes and to increase our willingness to engage in the challenges of managing it. Example: if my blood sugar is 217, I could think about how awful that is and beat myself up thinking about how it should be better and ruminate about how I keep screwing up my diabetes control. That’s probably just going to get me frustrated, depressed, and on my way to feeling overwhelmed/burned out though, and, ironically, might lead to less willingness to test next time if I know that if it’s a “bad” blood sugar, I’m going to go through all of that. I might end up avoiding testing as a result.” (A thread about Meditation).

#### Forum users’ attitudes toward the use of CAM for diabetes

Many forum users expressed positive attitudes toward using CAM for diabetes. This was apparent mostly with Ayurveda followed by herbal medicine and homeopathy (Table 2). It was often mentioned in the forums that CAM can cure diabetes in contrast to conventional medicine.

“You said, diabetes once set, can't be reversed. This is NOT the truth- as you see the UK homeopath cured himself, and the Hpathy article talks about 15 % cured cases in the literature. You don't know who could be one of the lucky ones who are cured, and even if not totally cured well worth it to have reduced the need for medicines.” (A thread about homeopathy).

However, posts about negative attitudes toward CAM were more frequent than posts about positive attitudes. Herbal medicine ranked at the top of the frequency of quotes related to negative perceptions followed by chiropractic (Table 2). Forum users were sceptical and dismissive of the portrayed benefits of CAM for diabetes. A patient explained how it is not feasible to replace insulin injections with acupuncture needles. Suspicions about claims that CAM can cure diabetes were also expressed.

“I am pretty confident that nothing a chiropractor can do will cause the pancreas to start working again. I am also doubtful that anything they do can even change bg levels or insulin needs, no matter how "centered" they make your body.” (A thread about chiropractic).

There were posts about having neutral attitudes towards CAM use. Neutral attitudes were most frequent with herbal medicine followed by acupuncture (Table 2). Forum users’ attitudes were mostly that they are open to try CAM as they have nothing to lose or trying CAM would not do any harm.

“I think we all know it wouldn't be this easy, no magic bullet, but maybe part of a therapy that would work is all I am suggesting”. (A thread about Yoga).

#### Experiences associated with conventional medicines as a factor in forum users’ perceptions of CAM

Forum users explained that the reason that drove them to use CAM is that using conventional medicine, especially insulin injections, will have a negative impact on their quality of life. Doubts about the benefits of conventional medicine were expressed. Forum users posted about how conventional medicines do not work for everyone. Disappointment was also expressed that conventional medicine has no cure for diabetes and CAM could offer that cure.

“Diabetes is the silent killer disease. There is no cure from its roots to the allopath science. Many peoples are dies in world by the diabetes and its related ailments. However, Ayurveda and Panchakarma have total cure for these” (A thread about Ayurveda).

#### Forum users convey their healthcare providers’ attitude toward CAM

Forum users rarely mentioned the views of their healthcare providers about the use of CAM for the management of diabetes when posting on online forums (Table 2). It was mentioned a few times that healthcare providers had not heard about the CAM type they were discussing with them regardless of how qualified and experienced their provider is.

“I have been taking berberine for four or five years with good results. During that time, I have filled out "what medicines do you take?" for probably 20 doctors. Only one had ever heard of berberine” (A thread about herbal medicine).

### Seeking other users’ experience with using CAM for treatment of diabetes

#### Seeking information

Online discussion forums seem to be an important mechanism for the forum users to ask about past experiences with CAM and seek information about the use of CAM for diabetes. Forum users also seem to seek information specific to the benefits of CAMs, frequency of use, or how long CAM takes to be effective.

“It had never occurred to that sticking needles in one self could be beneficial for diabetics. LoL. Has anyone tried it? ” (A thread about acupuncture).

Information seeking was most frequently mentioned for herbal medicine in the context of other types of CAM as it was noted 9 times (Table 2).

#### Seeking or giving instructions about how to ‘properly’ use CAM

Forum users seemed to seek information from previous users on how to use CAM ‘properly’. In addition, people who used CAM often volunteered to explain what they think is the proper way to use it. CAM users often replied to the original enquirer who said that they did not get the required result by CAM by attributing that to improper use of CAM and then they tried to explain how to use CAM properly. Some users also advised others of harmful ingredients and safety implications where CAMs were not used in moderation. This subtheme was most frequent for homeopathy, which emerged 6 times (Table 2).

“You need to take the right cinnamon. The kind commonly found in grocery stores, Cassia cinnamon, has a higher amount of a toxin called coumarin, which is hard on your liver. When you take amounts that simply season your food, you can metabolize it alright; but when you take therapeutic amounts for blood sugar, you ingest potentially unsafe amounts. Do some searching and make sure you take Ceylon cinnamon. It has barely a trace of coumarin. And yes, it does work to lower blood sugar. ” (a thread about cinnamon).

### Perceived effectiveness of CAM

#### Experience of CAM use

Unlike posts about attitude towards CAM, where posts about negative attitudes toward CAM were the most frequent among other attitudes, posts about experiencing positive results from using CAM were the most frequent, i.e. rather than negative results or no results (Table 2). Posts showed associations between using berberine instead of metformin due to metformin side effects. Berberine is a compound that is extracted from the barberry plant [22]. The reported effects of CAM were mostly lowering blood glucose and preventing morning spikes. Additionally, it was reported that patients use CAM to cope with diabetes.

“It helps me to be able to step back, examine my feelings. Mediation helps me to get a better perspective on what is my reality at that time and be able to accept it. For me this reduces the stresses so I can cope with my challenges.” (A thread about meditation).

The least frequent posts related to CAM results were posts related to experiencing negative results from CAM. Forum users experienced what one described as ‘disastrous’ effects of CAM on their diabetes, others posted the condition was deteriorating as a result of using CAM. Negative results of using CAM as experienced by forum users were hypoglycaemia, hyperglycaemia and what one described as a roller-coaster of blood glucose levels. Details about these side effects’ communications are described below under the forum users’ perception of CAM safety theme.

“I test my blood sugar prior to leaving and it's usually around the normal range between 100-130. I do the Yoga, sweat like crazy and come home and test again and my BS has consistently been around 200-260. This seems crazy because whenever I do aerobic exercises, I'm able to drop my BS down to a normal range.” (A thread about yoga).

Forum users posted about trying CAM for diabetes and experiencing no effects. The effect of CAM was measured by seeing if there were no effects on their blood levels, or their insulin needs did not change while using CAM. A patient used CAM for the purpose of curing diabetes and judged the effect based on the fact that CAM did not cure their diabetes.

“My mom tried EVERYTHING to "cure" my diabetes when I was younger. I went along with the idea up til I was around 15 to "cure" the diabetes. by that time I found nothing "cured" diabetes.” (A thread about Ayurveda).

#### Forum users experience an effect of CAM on diabetes while using CAM for other reasons

A few users mentioned having an unexpected effect on their diabetes while using CAM for other reasons. One forum user used massage for sore muscles and ended up having unexpected hypoglycaemia, and another forum user used chiropractic and noticed a reduction in his insulin needs.

### Forum users’ perception about the evidence base of CAM effectiveness

#### Forum users’ views on scientific studies about the effectiveness of CAM for diabetes

Forum users discussed studies on CAM effectiveness that they came across. They suspected that the results from those studies cannot be replicated and therefore cannot be used as evidence about the effectiveness of CAM on diabetes. Countries were referenced where a specific type of CAM is popular and this was linked to the prevalence of diabetes being still high despite the fact that it is used more frequently in that country.

“Chinese Traditional medicine doesn't seem to be helping the type 2 diabetes epidemic in China (they have surpassed the US in % of instances” (A thread about acupuncture).

Forum users used online discussion forums to share links to studies that substantiate the effectiveness of CAM on diabetes. They also talked about the studies they read or articles they came across that are about the effectiveness of CAM on diabetes. One user posted about how Harvard Medical School required its medical students to take a semester of homeopathy as a supporting argument that homeopathy is starting to get medically recognized.

“I just recently started taking berberine. I came across an article about a week ago. What have I got to lose?” (A thread about herbal medicine).

#### Forum users giving explanations about CAM effectiveness

Forum users used online discussion forums to add their inputs on how CAM works to manage diabetes. These explanations included that CAM corrected the metabolism or CAM contained soluble fibres that have a blood glucose lowering effect by slowing digestion and absorption. Another explanation was that CAM helps with eating less fried and sweet food as well as acting like an antitoxic, which helps to activate the cells therefore allowing insulin to work on cells. Stimulating the pancreas with yoga was one of the explanations of the effectiveness of CAM. A forum user posted that CAM helps with relaxing and reliving stress which leads to lowering the stress hormone therefore lowering insulin needs or was simply a placebo effect.

### What are the forum users' perceptions on the safety of CAM or/and conventional medicine

#### Forum users’ perceptions on CAM safety

Many forum users mostly viewed CAM as a safe form of therapy. They described it as harmless and potentially beneficial for treating diabetes. Experiencing no downside related to CAM use was also reported.

“To me it falls into the unlikely to cause any harm category, so it might be worth giving it a try” (A thread about herbal medicine).

Forum users expressed that they could not tolerate metformin due to its side effects. They resorted to using CAM for the management of diabetes solely to avoid metformin side effects. In a post those side effects were described as Metformin Moments or "Metformin Zombie". It was described how metformin side effects have a negative impact on patients’ quality of life.

“Metformin did not have any effect on her blood glucose. She only got bad side effects. As her husband, I have no printable things to say about Metformin and the doctor's response to what it did to her. Practically a year of her life was wasted to the zombie effect” (A thread about herbal medicine).

#### Forum users use online forums to discuss CAM side effects versus conventional medicines

A forum user described how he had stomach issues while using herbal medicine and another replied that that CAM type was killing parasites that already existed in his stomach and that is why he had those stomach issues. In discussing another side effect of CAM, a forum user complained about irregular sleep and high fasting glucose numbers while using acupuncture; another user replied that it is not possible to tell if the high fasting glucose numbers are caused by CAM or sleeping issues. Other side effects reported included hypoglycaemia and vomiting.

“My wife is type 2. I got her a massage for Christmas two years ago because she loves them. But we think she ended up with a deep tissue massage which she didn’t want. She ended up throwing up before the massage was even half done. They blamed it on her being diabetic.” (A thread about herbal medicine).

### CAM as an additional or replacement treatment for diabetes

#### Forum users' use or views on CAM as a complementary therapy

Forum users posted about their experiences with using CAM together with conventional medicine. Their experiences varied between seeing no additional results from adding CAM to their treatment, to experiencing an effect which they measured by alternating skipping one form of treatment at a time and noticing the change in blood glucose levels. A patient in one post wondered if using CAM along with conventional medicine would have a synergistic effect.

“I don't know if the 2 together give you double the effect?” (A thread about herbal medicine).

#### Forum users’ use or views on CAM as an alternative therapy

Forum users posted about their thoughts and experiences about using CAM for the management of diabetes instead of conventional medicine. They either used CAM instead of conventional medicine as they viewed both approaches as being supposed to have the same effect, or were considering stopping conventional medicine to use CAM alone because they were starting to see an effect of CAM. One user posted about experiencing abdominal side effects when using conventional medicine together with CAM and decided to use CAM alone.

“Obviously no one only you can make the decision about your tablets but I just couldn’t stand the pain I was in. and it looks as if you were having the same problems. I also was going to go to A+E but decided on a couple of day's trial without the tablets. Aloe Vera was on one of the other threads but can't remember which one, so I decided to give it a try. I've been off the tablets for about 4 weeks now and feel great. And haven’t had a bad reading since, but as I said I did tell the doc I wasn’t happy with them, but I didn’t say anything about the Aloe I just started taking that myself after reading the good reports.” (A thread about Aloe vera).

### Economic issues around the use of CAM for the management of diabetes

#### Access and cost related issues from the perspective of forum users

Forum users posted about wanting to use CAM but the cost of CAM was the reason they did not use it as insurance companies do not cover CAM treatments or conventional medicine is cheaper than CAM.

“When I needed diabetes meds, I considered it, but cost kept me away. My metformin was only $1.99 for a 3 month supply” (A thread about herbal medicine).

#### Recognition of CAM by medical insurers

Forum users posted about how insurance companies do not want recognition for the effectiveness of CAM as it will drive people away from orthodox treatment. They said that if CAM becomes more popular, insurance companies will no longer exist as the reason for their existence what was described by a forum user as “absurd health care costs”. Forum users also blamed pharmaceutical companies for the limited research and development in the field of CAM as such research would not be likely to generate revenue for the companies. Regarding CAM, forum users also suspected that commercially available CAM products are just made to exploit forum users to make money from them and do not actually work for diabetes.

“I would like to know whether a proper cure for Diabetes Type I and II is going to be reality in coming years. As I can see we have genome sequence, we have identified gene, SNPs in the gene and then proteins which are switched on or off due to this. What else is needed to find a cure? Or is it as they say "There is cure but no one wants to kill the golden goose”” (A thread about Ayurveda).

## Discussion

### Statement of key findings

This study set out to explore patients’ beliefs, experiences and preferences in relation to their use of CAM in diabetes through the use of existing data in online patient forum discussions. It also aimed to identify factors that attract patients with diabetes to use CAM or drive them away from conventional medicine into using CAM as well as diabetic patients’ positive and negative impressions of CAM use for the management of diabetes.

Our findings indicated that patients use online forums to seek information from previous users about the benefits of CAMs, frequency of use, how long CAM takes to be effective or how to use CAM ‘properly’. Forums users talked about the negative impact their condition or its management routine had on their quality of life, making them feel stressed, frustrated or overwhelmed about managing diabetes and how this was linked to their decisions to use CAM. Forum users used online forums to share links to studies that substantiate the effectiveness of CAM on diabetes or to add their inputs on how CAM works to manage diabetes. Access and cost related issues from the perspective of forum users and recognition of CAM by medical insurers were one of the subjects that were often discussed by forum users. Forum users often pointed to lack of ability of conventional medicines to cure diabetes as the reason to seek alternative management approaches in a hope to find a permanent cure.

### Strengths and Weaknesses

There is a dearth of qualitative research on diabetic patients’ use of CAM and there are limitations in existing studies. For example one study was limited to herbal medicine and it only recruited participants from one single community, Indian and Pakistani migrants. That study was also limited to a narrow context which was to find out if migrants still use CAM from their previous communities [23]. Another study only recruited participants with type 2 diabetes and discussed only limited CAM types [24]. This study adopted a rigorous search strategy that used the most popular CAM types among patients with diabetes as it was guided by the results of a global systematic review in order for the study scope to be as comprehensive as possible [6]. Issues such as hidden concerns about conventional medicine, for example metformin side effects, diabetic patients’ explanations of the effectiveness of CAM and issues related to medical insurance etc. were never brought up by any previous studies and have been identified by this study. Moreover, the fact that this study explored data already available online and not collected from patients in formal research settings is likely to be able to explore what patients with diabetes actually think as their opinions and concerns were expressed freely in a non-restrictive manner [12].

This study has some limitations. For example, it is not possible to promote validity of the data through member checking as users are anonymous and no consent are available to follow up those who identify themselves. In addition, the data included in this study is limited to the subjects discussed in the forums. It was not feasible to probe any further on any part of the data to gain a more in-depth view on any given subject. As there was no researcher involvement in this study to generate the data, and most comments analysed were in response to other forum users’ questions, the data may be under-representative of those with limited literacy including digital literacy. Even though the data were not restricted to any particular group of patients, information about people posting on the forums such as age, condition status of the patient and duration of the condition are limited. Moreover, forums are often vulnerable to unsolicited and covert advertising campaigns by product manufactures and there is no reliable way to ensure the validity of each individual post. Lastly, the study was limited to English language forums only and did not include forums in other languages.

### Interpretation

Previous studies that utilized data from patient online forums have often used selective lists of online forums [13, 14]. Our study explored all relevant forums identified through the comprehensive web search. In addition, the use of both content analysis and thematic analysis allowed key themes and subthemes to be identified and further described using relevant quotes.

The study has implications for clinical practice in promoting our ability to understand issues related to CAM safety, effectiveness and medication adherence as perceived by patients. The findings could be utilized by healthcare providers to further engage with the patients and promote rational self-care practices including the use of CAM.

### Future research

The results of this study have highlighted the importance of engaging with patients and exploring in-depth their use of CAM through further qualitative study and research in natural settings. Perspectives of healthcare professionals regarding their patients’ use of CAM is also important to enable them to engage more effectively with their patients. Further research on what makes patients resort to online forums to seek advice and share and discuss their condition rather than discussing the same with their healthcare professionals is important to promote better patient-provider interactions. Decision to use CAM as the standalone therapy and discontinuation of prescribed treatments due to perceived lack of effectiveness and greater side effects is likely to lead to negative health consequences for patients. Given the plethora of treatment options available in the management of diabetes, there is a need to develop and implement interventions focused on patients and healthcare professionals to promote patient-centred optimisation of prescribed treatments in diabetes. As online health information is constantly changing, online forums can further be utilized in research by regular analysis of their contents in order to gain a wider view and be able to identify all new aspects of diabetic patients’ management behaviours and concerns. Given their accessibility and expertise around wide range of over-the-counter products [25–27], research to promote better utilisation of community pharmacists for CAM related information can promote their rationale use.

This study was limited to data that were available through patient online forums and did not include similar discussions that might be available on social media platforms. However, such posts are worth researching in future studies.

## Conclusion

This study demonstrates that patients with diabetes use online forums to seek and offer advice around CAM use. There is a scope for professional societies, patient charities and health systems to offer such online platforms to offer support and provide evidence-based information to patients. Healthcare professionals should address patient concerns and queries regarding optimisation of prescribed medicines as some forum users described resorting to CAM use due to the side effects and lack of effective communication with healthcare professionals. Results should be interpreted with caution given limitations around reliability and trustworthiness related to data from online forums.
